# Blue-conversion of organic dyes produces artifacts in multicolor fluorescence imaging†

**DOI:** 10.1039/d1sc00612f

**Published:** 2021-05-18

**Authors:** Do-Hyeon Kim, Yeonho Chang, Soyeon Park, Min Gyu Jeong, Yonghoon Kwon, Kai Zhou, Jungeun Noh, Yun-Kyu Choi, Triet Minh Hong, Young-Tae Chang, Sung Ho Ryu

**Affiliations:** Department of Life Sciences, Pohang University of Science and Technology Pohang 37673 Republic of Korea sungho@postech.ac.kr genesis@postech.ac.kr; Integrative Biosciences and Biotechnology, Pohang University of Science and Technology Pohang 37673 Republic of Korea; Department of Chemistry, Pohang University of Science and Technology Pohang 37673 Republic of Korea

## Abstract

Multicolor fluorescence imaging is a powerful tool visualizing the spatiotemporal relationship among biomolecules. Here, we report that commonly employed organic dyes exhibit a blue-conversion phenomenon, which can produce severe multicolor image artifacts leading to false-positive colocalization by invading predefined spectral windows, as demonstrated in the case study using EGFR and Tensin2. These multicolor image artifacts become much critical in localization-based superresolution microscopy as the blue-converted dyes are photoactivatable. We provide a practical guideline for the use of organic dyes for multicolor imaging to prevent artifacts derived by blue-conversion.

## Abstract 2

Blue-conversion, a photooxidative conversion leading to the hypsochromic shift of absorption and emission spectra, occurs in popular organic dyes under conventional laser illumination and produces severe artifacts in multicolor fluorescence imaging.

## Graphical abstract

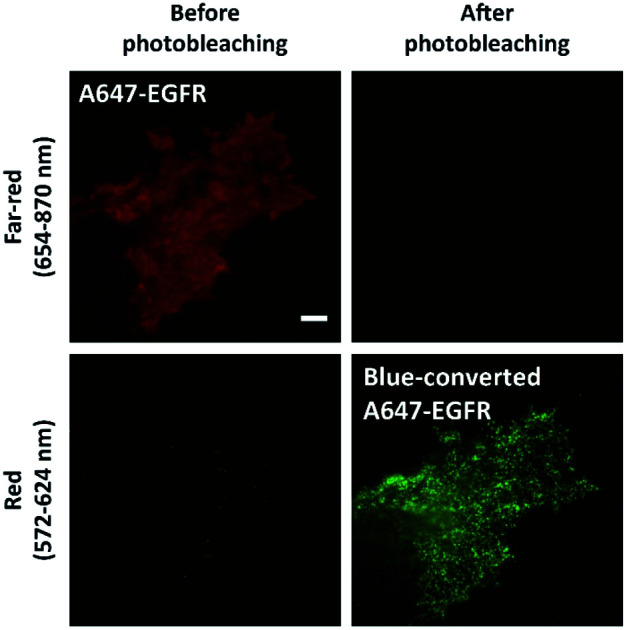

Biomolecules act in concert within subcellular structures to generate various biomolecular processes. Multicolor fluorescence imaging by labeling biomolecules with several fluorophores allows the visualization of the spatiotemporal relationships among them, providing direct evidence for understanding their concerted behaviors and functions.^1^

Choosing suitable dyes based on the purpose of the experiment is critical for fluorescence microscopy. Multicolor imaging requires fluorophores that fluoresce at distinct and well-separated spectra.^2^ Superresolution localization microscopy requires photoactivatable/-switchable fluorophores to achieve the blinking necessary to distinguish individual fluorophores within the diffraction limit.^3,4^ Long-term imaging requires highly photostable yet sufficiently bright fluorophores.^5^

Organic dyes are commonly utilized due to their advantageous characteristics, including a broad range of excitation and emission spectra, high brightness and photostability, and photoactivatable/-switchable properties.^4,6,7^ The specific labeling of a target protein with an organic dye has been achieved by using the antibodies crosslinked *via N*-hydroxysuccinimide ester or maleimide modified organic dyes or the genetic fusion of SNAP- or Halo-tags covalently binding to benzylguanine or chloroalkane moieties attached to organic dyes.^8–10^ The cytosolic proteins in live cells have been labeled with membrane permeable organic dyes.^11^

While investigating cell motility dynamics mediated by the epidermal growth factor receptor (EGFR) using live-cell fluorescence microscopy, we observed the unexpected phenomenon that organic dyes are converted to another species emitting shorter wavelengths than their original emission wavelength after the imaging. We labeled SNAP-tagged EGFR expressed on COS7 cells with O^6^-benzylguanine (BG)-conjugated Alexa Fluor 647 (A647). After imaging the A647-EGFR on the plasma membrane using total internal reflection fluorescence (TIRF) microscopy through the far-red channel (654–870 nm) with 642 nm laser illumination, we spotted unanticipated fluorescence in the red channel (572–624 nm) upon excitation by 561 nm laser illumination, which did not exist before the imaging of the A647-EGFR. It was formerly reported that far-red cyanine dyes, including A647 and Cy5, contain near-red fluorescent impurities.^12^ However, the amount of this newly appeared fluorescence in the red channel was strongly correlated with the amount of photobleached A647-EGFR by 642 nm laser illumination (Fig. 1a). We also observed the same phenomenon when endogenous EGFR was labeled with the A647-conjugated anti-EGFR antibody (Fig. S1†). To confirm this phenomenon *in vitro*, nonconjugated A647 dissolved in DMSO was directly illuminated with a 642 nm laser to induce photobleaching on the bulk scale. Surprisingly, instead of becoming colorless as expected, the blue-colored dye turned pink after sufficient laser illumination (Fig. 1b). This product contained two major substances detectable at 280 nm, one of which absorbed 561 nm light (Fig. 1c). Neither substance absorbed 647 nm light, indicating that the photobleaching of A647 was complete. When the 561 nm-absorbing substance was illuminated with a 561 nm laser, red fluorescence was emitted (Fig. 1d), which coincided with the *de novo* fluorescence of A647-EGFR produced by 642 nm laser illumination in the red channel (Fig. 1a).

**Fig. 1:**
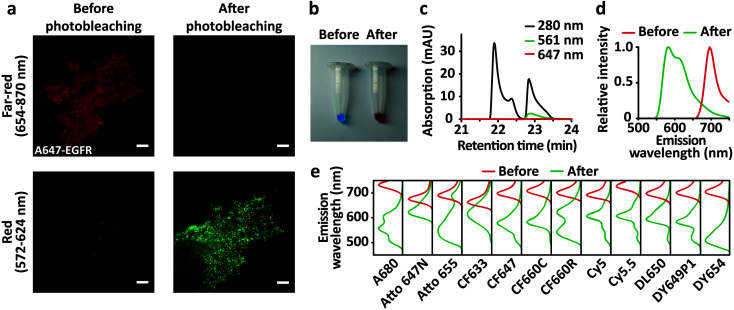
Blue-conversion of far-red organic dyes upon photobleaching. (a) TIRF images of A647-EGFR on COS7 cells in the far-red (upper panels) channel excited at 642 nm and the red (lower panels) channel excited at 561 nm before (left panels) and after (right panels) photobleaching of A647-EGFR. (b) A647 dissolved in DMSO before (left) and after (right) photobleaching using direct laser illumination. (c) HPLC analysis of A647 after photobleaching with absorbance at 280 nm, 561 nm, and 647 nm. (d) Emission spectra of A647 before (red) and after (green) photobleaching, excited at 647 nm and 561 nm, respectively. (e) Emission spectra of far-red organic dyes before (red) and after (green) photobleaching. Scale bars, 5 μm.

A few fluorophores based on coumarin and triarylmethane were previously reported to exhibit this ‘blue-conversion’ phenomenon derived by laser excitation,^13,14^ which can display residual crosstalk between multicolor channels in STED microscopy.^15^ However, blue-conversion of cyanine-based fluorophores remained controversial: while A647 was photoconverted to near-red upon intense and prolonged illumination with green/yellow lasers,^16^ A647 was one of the far-red organic dyes that were not turned into near-red upon two-photon excitation.^17^ Thus, we extensively explored whether blue-conversion derived by an excitation laser as we observed in A647 also occurs in commonly used and commercially available organic dyes in various chemical families for conventional fluorescent microscopy (Table S1†): cyanine (Alexa Fluor 647, Alexa Fluor 680, CF647, CF660C, Cy5, Cy5.5, Cy3B, Dylight 650, and Dyomics 649p1), benzopyrylium hemicyanine (Dyomics 654), rhodamine (CF633, CF660R, and silicon rhodamine), carborhodamine (Atto 647N), and oxazine (Atto 655). All these far-red dyes remained opaque and changed colors after direct illumination with the 642 nm laser on a bulk scale, except for initially colorless SiR (Fig. S2†). The color-changed dyes contained one or more species exhibiting distinct absorption and emission spectra at shorter wavelengths than those of the original species (Fig. 1e and S3†). Interestingly, we observed that the two cyanine dyes close to near-IR, Cy5.5 and A680 exhibited their blue-converted species with emission peaks at 607 nm and 603 nm, respectively, which are almost the same as the blue-converted species of their precursor far-red dyes, Cy5 and A647 with emission peaks at 607 nm and 611 nm, respectively (Fig. 1d, e and Table S1†). We also found that the emission peaks of blue-converted cyanine dyes are less blue-shifted as their aromatic rings are sulfonated, whereas those peaks are greatly blue-shifted as their rings are PEGylated. The blue-shifted emission peaks after the blue-conversion of Cy5 (no sulfonation and PEGylation), A647 (sulfonation), DY649P1 (sulfonation), CF647 (PEGylation) and CF660C (PEGylation) are at 59 nm, 54 nm, 49 nm, 86 nm, and 102 nm, respectively. A rhodamine, CF633, showed the lowest blue-shift of the emission peak after the blue-conversion (35 nm), while a benzopyrylium hemicyanine, DY654, showed the highest blue-shift (166 nm) among the dyes we examined. Compared to other chemical families, cyanines tended to display multiple blue-converted species. Blue-conversion of these far-red dyes was also confirmed *in vivo* (Fig. S4†). SNAP-EGFR was labeled to the dyes conjugated with the BG moiety, and the blue-conversion of all the examined dyes was observed, except for Atto 655 (Fig. S4†) although the strong blue-conversion of Atto 655 was displayed *in vitro* (Fig. 1e and S2†). This difference between *in vitro* and *in vivo* results might be due to the cleavage of BG-Atto 655 induced by the blue-conversion, making the fragments leave from the BG moiety conjugated to a SNAP-tag, which results in the loss of fluorescence for SNAP-EGFR after the blue-conversion. Furthermore, when Cy3B was photobleached by 561 nm laser illumination, *de novo* fluorescence appeared in the yellow channel (500–549 nm) upon 488 nm laser excitation (Fig. S4†), implying that the blue-conversion of the red dyes also widely occurs, including Lysotracker Red DND-99 (Invitrogen) that has been previously reported to exhibit red-to-green blue-conversion.^18^ Among the examined dyes with various chemical families, there is no dye that did not exhibit blue-conversion at all.

We quantitatively measured the degree of blue-conversion from the far-red to the red channel by estimating the number of dye molecules in each channel using their single-molecule intensities, which enables us to directly analyze the ratio of blue-converted molecules without determining the extinction coefficients of the unknown blue-converted species (see the details in Methods). The far-red dyes were blue-converted into the red channel by up to 5.37% (Fig. S5†). We could not observe the strong relationship between their chemical structures (or families) and the amount of blue-conversion (Table S1 and Fig. S5†).

Multicolor imaging has long been utilized in dichroic mirrors and emission filters to separate distinctly emitting fluorophores into desired channels. This technique relies on the fact that the excitation and emission spectra of fluorophores do not change during the imaging. However, this level of blue-conversion was expected to significantly invade the predefined spectral channels. To compare the multicolor fluorescence images produced by blue-conversion sensitive and resistant dyes, we selected A647 and CF660R. Although these fluorophores displayed similar levels of blue-conversion after complete photobleaching, CF660R is substantially more photostable than A647. Thus, the differential amount of blue-conversion of the two dyes can be proportionally induced by the duration of laser illumination.

We conducted the colocalization analysis of EGFR and Tensin2 as a case study. ErbB and Tensin families are previously known kinases and phosphatases involved in focal adhesion dynamics in cell motility.^19–21^ The direct association between ErbB and Tensin family members has been reported,^22,23^ but not between EGFR and Tensin2. BG-A647 or BG-CF660R was labeled with SNAP-Tensin2 coexpressed with red fluorescent protein (RFP)-conjugated EGFR in COS7 cells. Because we observed that red fluorescent protein (RFP) exhibited no blue-conversion, RFP was utilized to examine the effect derived by blue-conversion of far-red dyes only, although some fluorescent proteins were previously reported to exhibit the blue-conversion.^24–28^ Both A647- and CF660R-Tensin2 displayed patch-like structures (Fig. 2a and d) and RFP-EGFR showed a relatively even distribution over the entire plasma membrane (Fig. 2b and e), consistent with the previous reports.^29,30^ Interestingly, however, the distribution of EGFR gradually changed patch-like within 1 min of RFP-EGFR imaging in the cells coexpressing A647-Tensin2 (ESI Video S1†), whereas only the fluorescence intensity decreased while the distribution of EGFR did not change as expected in the cells coexpressing CF660R-Tensin2 (ESI Video S2†). This phenomenon occurred because the 561 nm laser used for exciting RFP also excites A647 and CF660R, causing differential levels of blue-conversion depending on their photostability. We confirmed that the signal for RFP-EGFR increased during the imaging only in the region where A647-Tensin2 was located (Fig. 2b and c). Because of its high photostability, the amount of blue-conversion of CF660R was not significant even after RFP was substantially photobleached (Fig. 2e and f). The two membrane proteins exhibited apparent colocalization on the plasma membrane when Tensin2 was labeled with A647 after the mild photobleaching that occurred under the conventional imaging conditions for fluorescence microscopy,^31^ but not when Tensin2 was labeled with CF660R (ESI Fig. S6†). It is possible to conclude that EGFR and Tensin2 are colocalized if a popular A647 was simply used, even though in reality these two proteins have no such relationship, as shown by using CF660R.

**Fig. 2:**
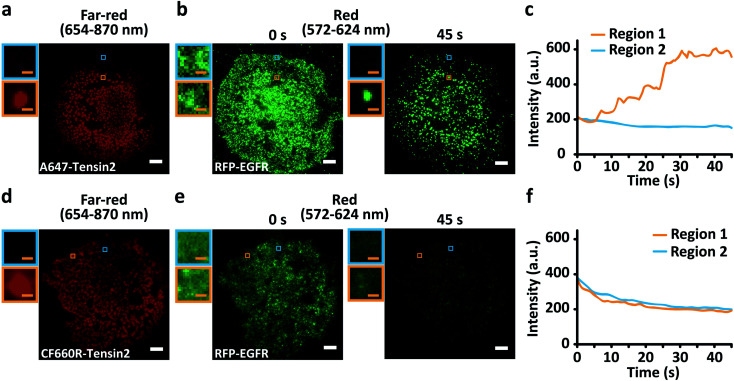
Multicolor imaging artifacts produced by blue-conversion. TIRF images of BG-A647- (a) or BG-CF660R- (d) labeled SNAP-Tensin2 in the far-red channel. TIRF images of RFP-EGFR (b and e) in the red channel with 45 s of continuous imaging. The insets display magnified images of the selected regions at the same positions with (orange box) or without (blue box) the Tensin2 signal in the far-red channel. (c and f) Time profiles of the fluorescence intensity in the selected regions. Scale bars, 5 μm (white) and 0.5 μm (orange). The fluorescence intensity in the selected regions for the red channel kept increasing during the imaging of RFP only in the region where A647-Tensin 2 was located whereas kept decreasing in the region where A647-Tensin 2 was not located (c). The fluorescence intensity in the selected regions for the red channel kept decreasing during the imaging of RFP regardless of regions where CF660R-Tensin 2 located or not (f).

We further observed that many organic dyes, including A647, A680, Atto 647N, CF633, CF647, CF660C, CF660R, Cy5, Cy5.5 DL650, DY649P1, and SiR, blue-converted by laser excitation initially existed largely in a dark state, which could be recovered to a bright state by UV illumination (Fig. S4a–c, e–l and n†). Because superresolution techniques based on single-molecule localization rely on the UV-induced photoactivatability of fluorophores, we explored the effect of blue-conversion on multicolor superresolution imaging. By replacing the RFP with mEos3.2 for the conjugation of EGFR, we performed colocalization analysis of EGFR and Tensin2 using single-molecule localization microscopy. The reconstructed superresolution image of mEos3.2-EGFR showed apparent patch-like structures colocalized with A647-Tensin2 at a subdiffraction resolution (Fig. 3a and b), whereas no significant colocalization was observed in the superresolution imaging with CF660R-Tensin2 (Fig. 3c and d). Not only the recovered amount of the blue-converted species in a bright state by UV photoactivation, but also the single-molecule brightness of the blue-converted species contributed to the production of the artifact in the reconstructed images. The blue-converted A647 was only slightly brighter than mEos3.2, making it almost impossible to distinguish between the two fluorophores. However, the blue-converted CF660R was significantly dimmer than mEos3.2, allowing the automatic rejection (SNR < 3) during the image reconstruction.

**Fig. 3:**
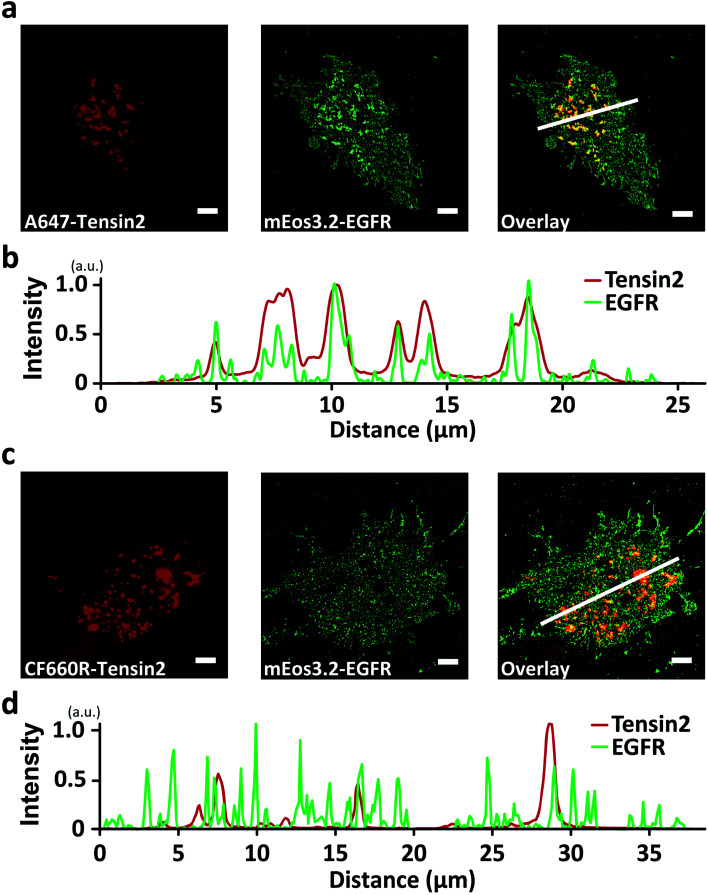
Multicolor superresolution imaging artifacts produced by blue-conversion. TIRF images of BG-A647- or BG-CF660R-labeled Tensin2 in the far-red channel (left panels in (a) and (c)). Reconstructed superresolution images of mEos3.2-EGFR in the red channel (middle panels in (a) and (c)). Overlay of the images from the two channels (right panels in (a) and (c)). (b) and (d) Intensity profiles of the indicated lines (white) in the overlaid images. Scale bars, 5 μm.

The demonstrated colocalization artifact derived by blue-conversion of organic dyes is an exemplary case where the expression level of Tensin2 is an order-of-magnitude higher than that of EGFR. Even below 2% of the blue-conversion can produce a detrimental artifact in multi-color biomolecule imaging in which the asymmetric amount of labeling between different channels is frequently encountered. Numerous cases with differential expression levels can be found in the human proteome.^32^ Furthermore, labeling efficiency varies from different targets and methods. For instance, the affinity of antibodies used to label biomolecules varies significantly from target-to-target and clone-to-clone. Besides, blue-conversion is accompanied by photobleaching of the labeled dye with exponential kinetics. Thus, not only the increase of blue-converted fluorescence but also the exponential decrease of the labeled dye reduces their signal gap. Finally, colocalization analysis depends on the local spatial correlation rather than the signal amplitude between the fluorescence detected in two channels. Thus, even the small bleed-through caused by blue-conversion can be critical in colocalization analysis if the targets exhibit spatial patterns.

Previously, it has been reported that triarylmethane dyes and coumarin dyes were blue-converted by photooxidative *N*-dealkylation.^13,33–35^ Furthermore, *N*,*N*′-di-*tert*-alkylrhodamine fluorophores have been recently developed to prevent the blue-conversion based on its mechanism.^14^ However, the chemical mechanism behind the blue-conversion of cyanine dyes (A647, A680, Cy5, Cy5.5, CF647, CF660C, DL650, and DY649P1) is not fully understood although a photooxidative reaction is essential to their blue-conversion (Fig. S7†). When we depleted oxygen using PCA/PCD, the blue-conversion of A647 significantly reduced (Fig. S7a†). In particular, singlet oxygen is likely involved in the blue-conversion process because the amount of blue-conversion showed a positive correlation when we modulated the level of singlet oxygen (Fig. S7b and c†). Recently, a study^36^ published during the revision of our manuscript reported that Cy5 becomes dioxetane species (em_max_ = 434 nm), photooxidative cleavage products (em_max_ = 210 nm, 345 nm, 370 nm), and Cy3 (em_max_ = 540 nm) after the blue-conversion, although the mechanism behind its blue-conversion has not been fully described. However, unlike their study, we observed that Cy5 produced the blue-converted species with their emission peaks majorly at 607 nm, 567 nm, and 523 nm (Fig. 1e). Especially, the blue-converted species at 607 nm was responsible for more than 50% of the emission spectrum of the blue-converted Cy5 (Fig. 1e) and this species was primarily detected both *in vitro* and *in vivo* in the red channel (572 to 624 nm) (Fig. S4†), but the recent study entirely lacks this principal species. Although the authors claimed that these species were not observed lower than 0.10 kW cm^−2^ where super-resolution microscopy works, our blue-conversion data for Cy5 were obtained after the bleaching at 0.05 kW cm^−2^ and clearly demonstrated the practical super-resolution imaging that the blue-converted species was detected in the red channel (Fig. 3). Interestingly, we found that Cy5.5 close to near-IR (em_max_ = 694 nm) and its precursor, Cy5 (em_max_ = 666 nm), exhibited their blue-converted species with the same emission peaks at 607 nm. Another case was also observed with another cyanine dye close to near-IR, A680 (em_max_ = 702 nm), and its precursor, A647 (em_max_ = 665 nm), the blue-converted species of which showed the emission peaks at 603 and 611 nm, respectively (Fig. 1d, e, and Table S1†). The modifications of Cy5.5 and A680 were introduced into their aromatic rings of the precursors while the length of their polymethine chain was preserved. Furthermore, other cyanine dyes with the same length of polymethine chains exhibited a similar range of emission peaks (579 to 632 nm) (Table S1†), implying that a primary factor contributing to the blue-conversion of cyanine dyes is the length of their polymethine chains. This interpretation is also supported by that Cy3B with the shorter length of the polymethine chain exhibited its blue-converted fluorescence mainly in the yellow channel (500 to 549 nm) (Fig. S4o†).

We provide a practical guideline for the use of organic dyes for multicolor fluorescence imaging to prevent artifacts derived by blue-conversion. First, unnecessary excitation of the dye should be minimized because it not only diminishes the original fluorescence intensity but also produces *de novo* fluorescence in other channels for shorter emission spectra. Second, highly photostable dyes should be adopted. If photosensitive dyes must be utilized for specific applications, an oxygen scavenging system should be included because oxygen is critical to the process of blue-conversion (Fig. S7†), although its use may limit live-cell applications. The photostabilizer-conjugated fluorophores or recently developed blue-conversion-resistant triarylmethane fluorophores could also be utilized.^14,37^ Third, when the expression levels of two proteins are substantially different, the protein with the higher expression should be labeled with a dye with a shorter wavelength or partially labeled to compensate for the expression level. We also provide a step-by-step procedure for checking and troubleshooting for the artifacts derived by blue-conversion of organic dyes in multicolor fluorescence imaging (ESI†).

Although we introduced the immediate negative effect of the blue-conversion of organic dyes, this new photoconversion pathway of cyanine dyes can bring the advantages of fluorescence imaging applications. The super-resolution techniques require the photoactivation (or photoswitching) of organic dyes where an oxygen scavenging system and a primary thiol are typically required to induce their photoactivatability, which possibly exerts an adverse effect in live cell imaging. However, the photoactivation of the blue-converted species occurs without requiring any buffer conditions as it occurs in water, PBS, and DMEM, aiding the super-resolution microscopy for live cells in their physiological conditions. Although we demonstrated the artifact of super-resolution images derived by the blue-conversion of A647 (Fig. 3a) as a limitation, this demonstration can be interpreted as an advantage of super-resolution microscopy using the blue-conversion of A647 working without requiring any buffer conditions.

Care must be taken in multicolor imaging applications, including colocalization, fluorescence resonance energy transfer, fluorescence correlation spectroscopy, single-particle tracking, or screenings using fluorescence-based multi-well plate format assays, to prevent false positives produced by blue-conversion of organic dyes.^38^ Controls are essentially required by performing each single labeling for all the multi-color channels utilized or using the blue-conversion resistant dyes to repeat the results. In addition to the blue-conversion, the red-conversion of organic dyes such as Hoechst 33258, DAPI, and Bodipy FL from green to yellow, orange, red or near-red has been previously reported,^39–41^ which also should be carefully considered to avoid the multicolor imaging artifacts.

## Methods

### Reagents

Benzylguanine-conjugated Alexa Fluor 647 (#S9136S) and benzylguanine-conjugated Dyomics 649P1 (#S9159S) were purchased from New England BioLabs. Alexa Fluor 647 (#A20006), Alexa Fluor 680 (#A20008) and DyLight 650 (#62265) conjugated with succinimidyl ester (NHS ester) were purchased from Thermo Scientific. Atto 647N NHS ester (#18373) and Atto 655 NHS ester (#76245) were purchased from ATTO-TEC. Cy3B NHS ester (#PA63101), Cy5 NHS ester (#PA15101), and Cy5.5 NHS ester (#PA15601) were purchased from GE Healthcare. CF633 (#92133), CF647 (#92135), CF660C (#92137), and CF660R (#92134) conjugated with NHS ester were purchased from Biotium. Dyomics 654 conjugated with NHS ester (#654-01) was purchased from Dyomics (Jena). Each dye with NHS ester was reacted with BG-NH2 (#S9148S, New England Biolabs) in anhydrous dimethylformamide (DMF, #227056, Sigma-Aldrich) at 30 °C overnight according to the manufacturer's instructions (New England Biolabs).

### Microscope setup

Multicolor imaging was performed on a homemade objective-type total internal reflection fluorescence (TIRF) microscope built on an inverted microscope (IX-81, Olympus) equipped with an XYZ automated stage (MS-2000, Applied Scientific Instrumentation). A 405 nm laser (DL-405-120, Crystal Laser), a 488 nm laser (35-LAL-415-220R, Melles Griot), a 561 nm laser (YLK 6150T, Lasos) and a 642 nm laser (2RU-VFL-P-1000-642, MPB Communications) were aligned with an oil-immersion TIRF objective lens (APON 100XOTIRF/1.49, Olympus). The fluorescence from multiple fluorophores was separated using a dichroic mirror (ZTUV-405/488/561/647RPC, Chroma) and emission filters (ET595/50m and T635lpxr, Chroma) and collected by two electron multiplying charge-coupled device (EM-CCD) cameras (iXon Ultra 897, Andor Technology) in an adaptor (TuCam, Andor Technology). A 1.6× amplifier and a 1.43× tube lens were used for higher magnification. All instrument operation and data acquisition were controlled by MetaMorph (Molecular Devices) and custom plug-ins written in MATLAB (MathWorks).

### Plasmid DNA

RFP-EGFR was kindly gifted by Dr Thorsten Wohland (National University of Singapore). To install Tensin2 at the N-terminus of SNAP, the SNAP-tagged gene from the pSNAPf vector (N9183S, New England Biolabs) was subcloned into pEGFP-C1/Tensin2 with the following primers 1–2. SNAP-EGFR and mEos3.2-EGFR were prepared as previously described.^38^

Primer 1: 5′-CGCTAGCGCTACCGGTCGCCACCATGGACAAAGACTGCGAA.

Primer 2: 5′-GGACTTCATTGAATTCTGTGAGGGACCCAGCCCAGGCTTGCCCAG.

### Sample preparation

COS7 cells (American Type Culture Collection, ATCC) were cultured in Dulbecco's modified Eagle's medium (DMEM, 12-604F, Lonza) supplemented with 10% (v/v) FBS (Gibco) at 37 °C, 5% CO_2_, and 95% humidity in a 6-well plate. The transient expression of SNAP-EGFR alone or the coexpression of SNAP-Tensin2 and RFP-EGFR or SNAP-Tensin2 and mEos3.2-EGFR was achieved by plasmid transfection using Lipofectamine LTX (#15338100, Invitrogen) according to the manufacturer's protocol. After 36 h, the cells were treated with 1 μM BG-organic dyes for 30 min and then washed three times with PBS. The cells were detached with 1 mM EDTA and then seeded onto a 25 mm glass coverslip in phenol red-free DMEM with 10% FBS. For cell fixation, the phenol red-free DMEM was removed, and the coverslips were rinsed with PBS. Then, the cells were fixed with 4% paraformaldehyde and 0.2% glutaraldehyde in PBS for 15 min at 37 °C and washed three times with PBS.

### Blue-conversion of organic dyes

For blue-conversion of organic dyes *in vitro*, the nonconjugated far-red organic dyes were dissolved in anhydrous DMSO or distilled water at 500 μM to 5 mM according to their solubility. Then, 100 μL aliquots of the dyes were transferred to e-tubes and directly illuminated with a 642 nm laser with a beam size greater than the size of the e-tube and a flux of 10 kW cm^−2^. The completion of blue-conversion required 24 h to 72 h depending on the photostability of the dye. Photographs of the original dyes were taken at 100 μM to match the color saturation of the blue-converted dyes for comparison under white LED light.

For blue-conversion of far-red organic dyes *in vivo*, BG-conjugated far-red organic dyes were utilized to label SNAP-EGFR expressed on COS7 cells. The labeled dyes on the cells were almost completely photobleached by the epi-illumination of the 642 nm laser at 50–500 W cm^−2^ within 10 s to 1 min depending on the photostability of the dyes until individual fluorophores could be resolved. Typically, cyanine dyes such as A647, A680, Cy5, Cy5.5, and DY654 were photobleached quickly, while rhodamine dyes or non-cyanine dyes such as Atto 647N, Atto 655, CF633, CF660C, CF660R, and SiR were photobleached slowly. For specific cases, DY654 was photobleached at 50 W cm^−2^ approximately for 10 s, whereas Atto 655 was photobleached at 500 W cm^−2^ approximately for 1 min. These ranges of laser power and exposure duration did not induce any noticeable damage to live cells. For photoactivation of the blue-converted organic dyes, the blue-converted organic dyes on the cells were illuminated through TIR using a 405 nm laser with an intensity of 0.6 W cm^−2^ for 20 s.

### Multicolor fluorescence imaging

SNAP-EGFR labeled with organic dyes on live (Fig. 1, S1, and S4†) or fixed (Fig. 2 and 3) COS7 cells was TIR illuminated using a 642 nm laser with an intensity of 0.2 W cm^−2^ and an exposure time of 500 ms in the far-red channel and using a 561 nm laser with an excitation intensity of 10–20 W cm^−2^ with an exposure time of 500 ms in the red channel.

For colocalization analysis, multispectral fluorescent beads (TetraSpeck Microspheres, 0.2 μm, Thermo Scientific) were imaged at the single-molecule level in both channels immediately after cell imaging. To register the images from the two EM-CCDs, an affine transformation matrix was calculated using the bead images.

### Fluorescence spectroscopy of the blue-converted organic dyes

The absorption spectra of the original and blue-converted organic dyes were measured using a NanoDrop 2000 (Thermo Scientific) according to the manufacturer's instructions. The emission spectra of the dyes were measured using a NanoDrop 3000 (Thermo Scientific). The wavelength of the absorption maximum of each dye was used as the excitation wavelength as follows: Alexa Fluor 647 at 561 nm; Alexa Fluor 680 at 527 nm; Atto 647N at 573 nm; Atto 655 at 474 nm; CF633 at 510 nm; CF647 at 497 nm; CF660C at 525 nm and 555 nm; CF660R at 594 nm; Cy5 at 564 nm; Cy5.5 at 540 nm; Dyomics 649P1 at 565 nm; Dyomics 654 at 510 nm; and DyLight 650 at 566 nm.

### HPLC analysis of the blue-converted organic dye

The liquid chromatographic separation of the blue-converted dyes was conducted using a C18 column (250 mm × 21.2 mm, 5 μm, Phenomenex) with an HPLC instrument (Prominence, Shimadzu Scientific Instruments). A gradient solvent system of 10 : 90 to 95 : 5 acetonitrile : water with 0.1% TFA was used with a run time of 60 min. The flow rate was 4 mL min^−1^ with a mobile phase consisting of 0.1% trifluoroacetic acid (TFA) in water and 0.1% TFA in acetonitrile under binary gradient conditions. The absorbance was monitored at 280 nm, 561 nm and 647 nm using a photodiode array system. The result of HPLC analysis is displayed in Fig. 1c.

### Quantification of the blue-conversion level

The measurement of the extinction coefficients of the blue-converted organic dyes for quantification was challenging because of issues associated with low yield and difficulty in the purification of the multiple blue-converted species derived from their original form. Instead, we used an alternative quantification method utilizing single-molecule intensity, which enables us to estimate the degree of blue-conversion from its original channel to the other channels without determining the extinction coefficients of each blue-converted species.

The total intensity of a far-red organic dye labeled on SNAP-EGFR on COS7 cells was measured in the far-red channel. Then, the labeled dye was photobleached up to the density at which individual fluorophores could be resolved. The average single-molecule intensity was measured from hundreds of individual fluorophores with the same imaging conditions to obtain a total intensity measurement. The absolute amount of the organic dye was calculated by dividing the total intensity by the single-molecule intensity. Then, this procedure was repeated for the blue-converted dye in the red channel. The level of blue-conversion was determined using the absolute number of dye molecules before photobleaching in the far-red channel divided by the absolute number of blue-converted molecules after photobleaching in the red channel.

### Superresolution fluorescence imaging

Glass coverslips (25 mm, #0111580, Marienfeld Laboratory Glassware, Lauda-Konigshofen) were cleaned by sonication in a water bath (1510R-DTH, Branson) with deionized water for 5 min, then in acetone (#A0097, Samchun Chemical) for 30 min, and in 1% hydrofluoric acid (#695068, Sigma-Aldrich) for 15 min. Then, the coverslips were rinsed 20 times with deionized water. Next, the coverslips were sterilized in ethanol (#1.00983.1011, Merck) under UV light for more than 30 min and washed three times with PBS. The coverslips were coated with fibronectin (#F2006, Sigma-Aldrich) (100 μg mL^−1^) dissolved in PBS for 1 h, prior to seeding the COS7 cells coexpressing mEos3.2-EGFR and SNAP-Tensin2 labeled with BG-Alexa Fluor 647 or BG-CF660R. After the seeded cells were attached and spread on the coverslip for 2 h, single-molecule localization microscopy was performed as previously described.^42^

## Data availability

All relevant data supporting the key findings of this study are available within the article and its ESI† or from the corresponding author upon reasonable request.

## Author contributions

D.-H. K. contributed conceptualization, methodology, investigation, analysis, manuscript preparation, funding acquisition, and supervision. Y. C. contributed conceptualization, methodology, investigation, analysis, and manuscript preparation. S. P. contributed conceptualization, methodology, investigation, and analysis. M. J. contributed investigation. Y. K. contributed investigation. K. Z. contributed investigation. J. N. contributed investigation. Y.-K. C. contributed investigation. T. M. H. contributed investigation. Y.-T. C. contributed resources. S. R. contributed funding acquisition and supervision.

## Conflicts of interest

The authors declare no competing interest.

## Supplementary Material

SC-012-D1SC00612F-s001

SC-012-D1SC00612F-s002

SC-012-D1SC00612F-s003
