# Supplementary material for: Blue-conversion of organic dyes produces artifacts in multicolor fluorescence imaging
Source: Chem Sci. 2021 May 18;12(25):8660–7. doi: 10.1039/d1sc00612f (PMC8246296; doi:10.1039/d1sc00612f)
Supplement: SC-012-D1SC00612F-s001 [file SC-012-D1SC00612F-s001.pdf]

Electronic Supplementary Material (ESI) for Chemical Science.
This journal is © The Royal Society of Chemistry 2021

A procedure for checking for the artifacts derived by blue-conversion of organic dyes in multicolor fluorescence imaging.

This procedure is to aid users to check for multi-color imaging artifacts derived by blue-conversion of organic dyes. Because a fluorescence microscope system can have different sets of lasers and filters, here we described lasers and channels as a conventional color-based terminology instead of wavelength. The excitation and emission spectra of blue-converted organic dyes are distinctly variable. Therefore, the amount of blue-conversion to each channel may vary according to user's microscope settings and organic dyes adopted for their applications. Nevertheless, this procedure can be generally applicable to fluorescence microscope systems with predefined spectral windows (lasers, dichroic mirrors, and emission filters with fixed wavelengths).

When a positive multi-color colocalization is observed by using organic dyes under fluorescence microscopy, controls are essentially required to confirm that the result is not a false positive derived by blue-conversion. The integrity of multicolor fluorescence images might be compromised by blue-conversion especially when the application requires high laser power, long-term imaging, blue-conversion sensitive dyes, or asymmetric level of fluorescently-labeled biomolecules in different channels.

1. Prepare an extra set of the samples used for multicolor fluorescence imaging experiments.

2. Label the samples with each dye alone for single-color fluorescence imaging.

3. Check if any fluorescence signal is observed in the shorter wavelength channels (other than the designated channel) in the same imaging condition used for multicolor fluorescence imaging .

4. Image the labeled sample in the designated channel under the same imaging condition used for multicolor fluorescence imaging.

5. Check the imaged sample for blue-conversion in the shorter wavelength channels (other than the designated channel) under the same imaging condition used for multicolor fluorescence imaging.

6. If a significant level of blue-conversion signal is observed, try the followed procedures for troubleshooting blue-conversion.

  6-1. Use a different dye pair to utilize a spare multicolor channel that did not exhibit the blue-conversion signal in the same imaging condition used for multicolor fluorescence imaging if available.

  6-2. Replace the dye with a blue-conversion resistant dye if available.

  6-3. Add an oxygen scavenging system to substantially lower the oxygen level in an imaging buffer if applicable.

  6-4. Label the sample partially with diluted concentration of the dye considering the level of blue-conversion signal.

  6-5. Change the labeling combination as a target with a higher expression can be imaged in a shorter wavelength channel.

7. Confirm the result in multicolor fluorescence imaging (whether a positive multi-color colocalization is still observed).

Fluorescent dye	Absorption (nm)	Emission (nm)	Blue-converted absorption (nm)	Blue-converted emission (nm)	Chemical family	Chemical structure	Ref
Alexa Fluor 647	650	665	551<br>511<br>395	611<br>579	Cyanine	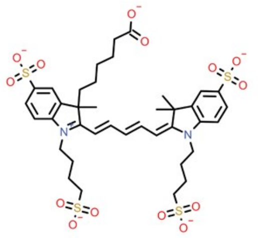	1, 3
Alexa Fluor 680	679	702	535<br>491<br>475<br>423	603<br>555<br>515	Cyanine	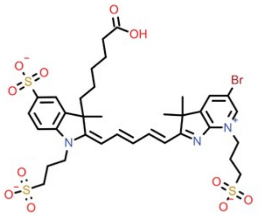	2
CF647	650	665	559<br>527<br>499<br>423	579<br>527	Cyanine	Structure not available. Cyanine-based CF dyes are produced by using PEG attachment to the aromatic rings.	4
CF660C	667	685	559<br>523<br>419	583<br>511	Cyanine		
Cy5	649	666	567<br>535<br>395	607<br>567<br>523	Cyanine	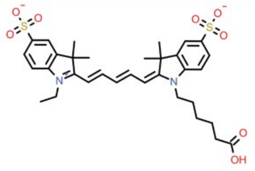	1, 3
Cy5.5	675	694	583<br>535<br>427<br>395	607<br>567<br>523	Cyanine	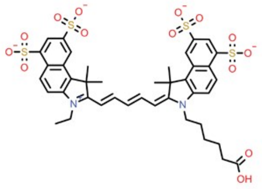	2
Cy3B	559	570	Not Available	Not Available	Cyanine	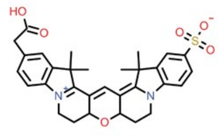	1
Dylight 650	652	672	567<br>535<br>503<br>423	595<br>519	Cyanine	Structure not Available	-

Dyomics 649P1	654	672	563<br>535<br>479<br>423	623<br>583<br>519	Cyanine	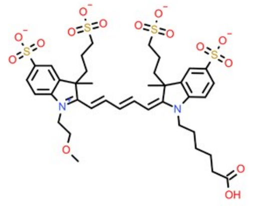	5
Dyomics 654	653	677	423	511	Benzopyrylium hemicyanine	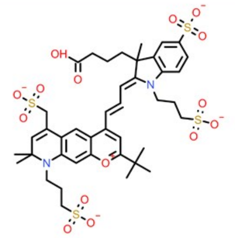	3
CF633	630	650	591<br>535<br>491<br>395	615<br>511	Rhodamine	Structure not available. Rhodamine-based CF dyes are produced by using an imidazole substitution of the benzene ring.	4
CF660R	663	682	591<br>395	623<br>543	Rhodamine		
Silicon rhodamine	652	674	Not Available	Not Available	Rhodamine	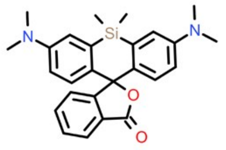<br>Non-fluorescent<br>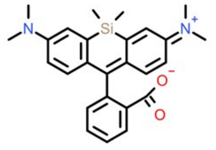<br>Fluorescent	6
Atto 647N	646	664	567<br>535	623	Carborhodamine	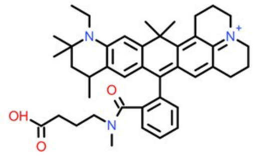	3, 7
Atto 655	663	680	395	547	Oxazine	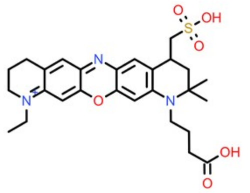	1

Table S1. The chemical information of organic dyes used in this study.

The absorption and emission wavelengths of the organic dyes before and after blue-conversion are provided. The chemical family and structure of the organic dyes are provided when available[1-3, 5-7]. The

chemical structures of the cyanine-based or rhodamine-based CF dyes are not released, but their key structural designs are described by the vendor[4]. The chemical structure of cyanine-based Dylight 650 is not released.

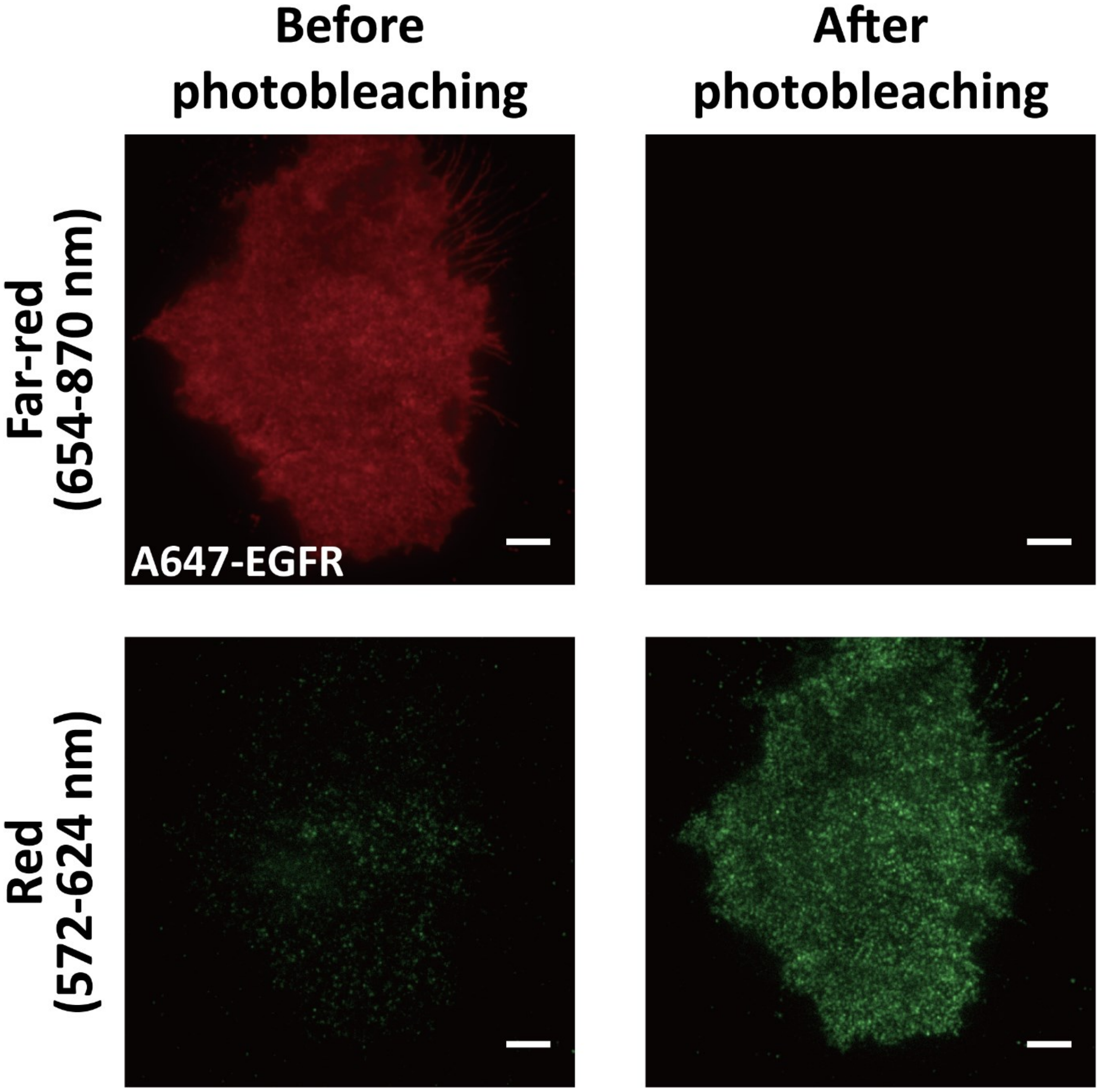

Supplementary Fig. 1 Blue-conversion of A647-conjugated anti-EGFR antibody labeled on EGFR

TIRF images of A647-conjugated anti-EGFR antibody labeled on SNAP-EGFR in the far-red channel excited at 642 nm (*upper panels*) and the red channel excited at 561 nm (*lower panels*) before (*left panel*) and after (*right panel*) photobleaching of A647. Scale bars, 5 µm.

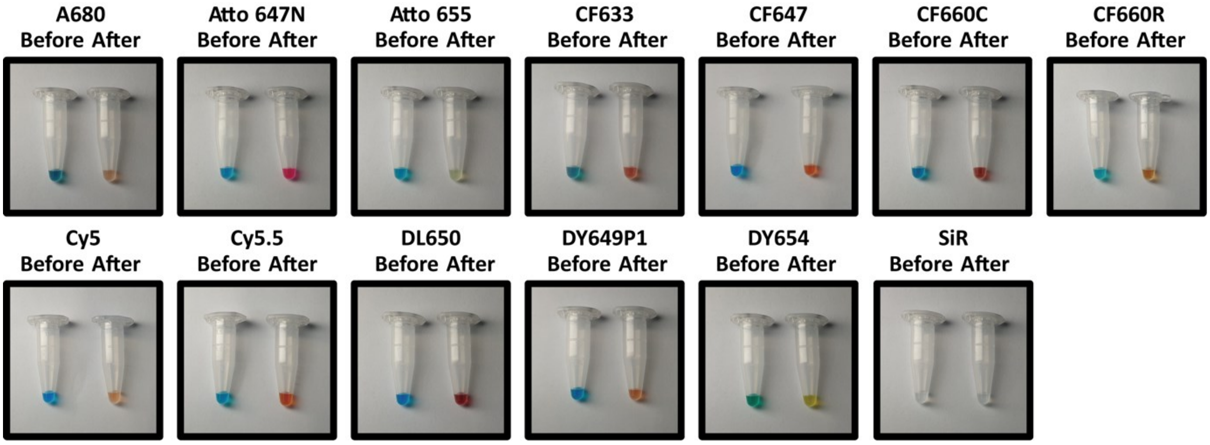

Supplementary Fig. 2 Blue-conversion of far-red organic dyes *in vitro* by direct laser illumination

Photos of A680, Atto 647N, Atto 655, Cy5, Cy5.5, CF633, CF647, CF660C, CF660R, DL650, DY649P1, DY654, and SiR before (left) and after (right) photobleaching at a bulk scale by direct illumination with a 642-nm laser.

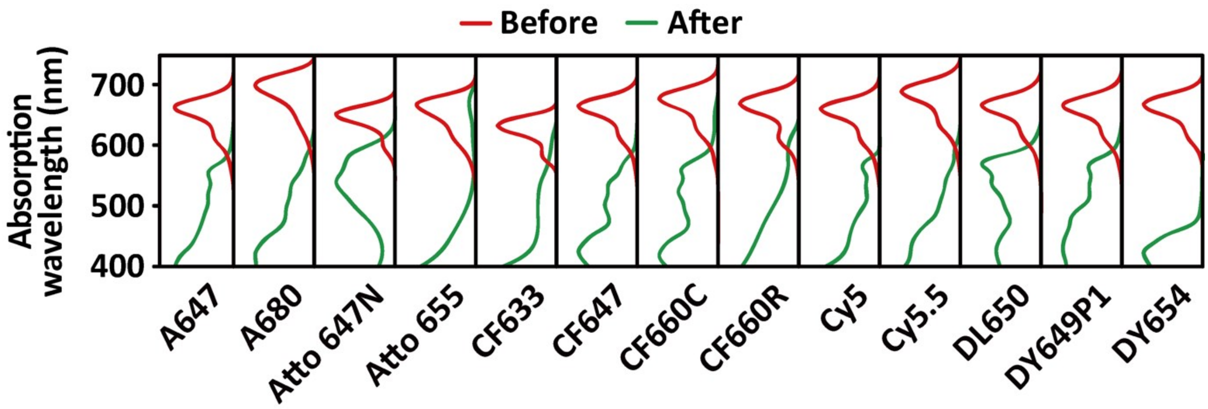

Supplementary Fig. 3 Absorption spectra of far-red organic dyes before and after photobleaching

Absorption spectra of A647, A680, Atto 647N, Atto 655, CF633, CF647, CF660C, CF660R, Cy5, Cy5.5, DL650, DY649P1, and DY654 before (red) and after (green) photobleaching at a bulk scale by direct illumination with a 642-nm laser.

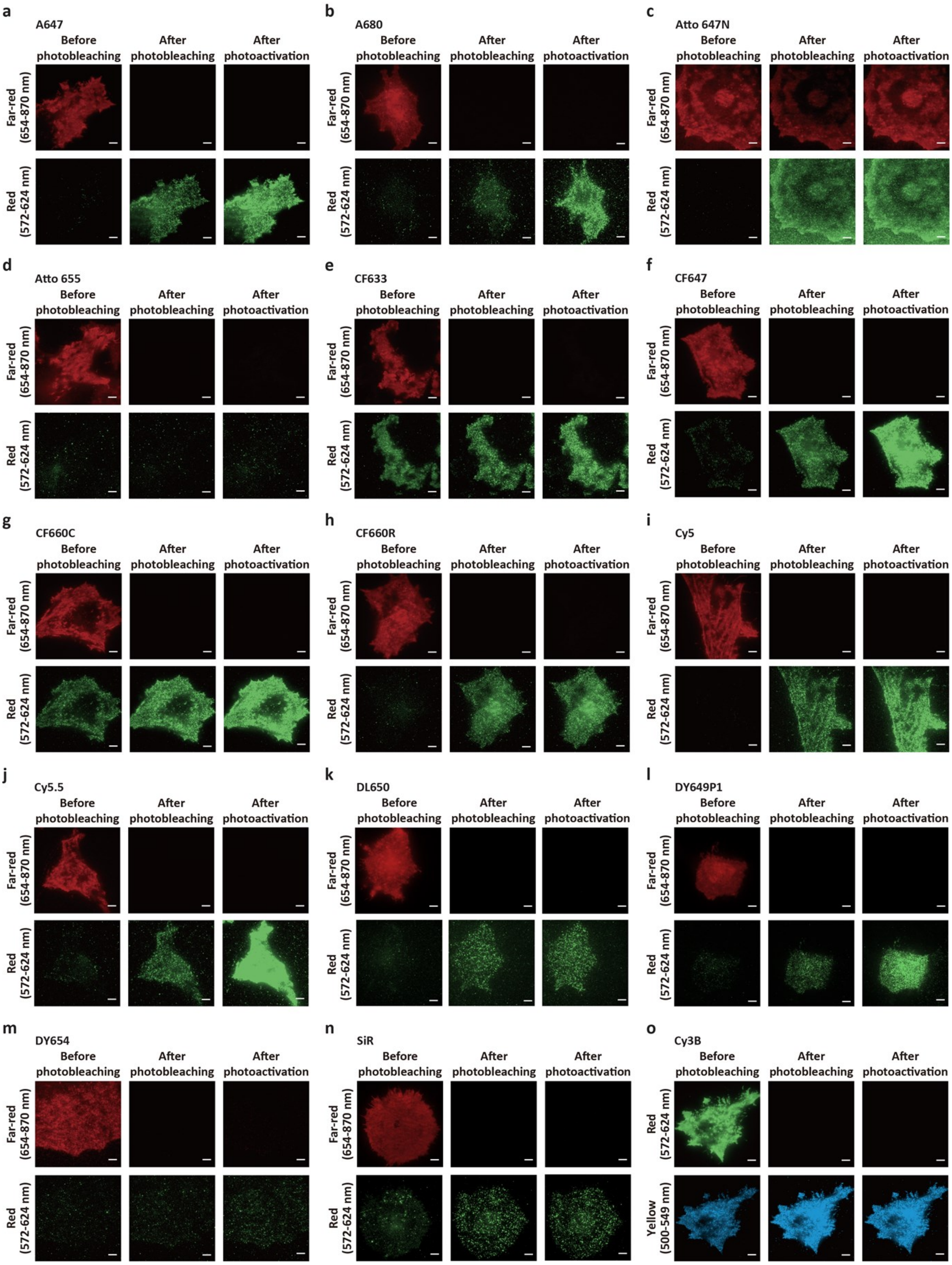
a
A647
Before photobleaching
After photobleaching
After photoactivation
Far-red (654-870 nm)
Red (572-624 nm)
b
A680
Before photobleaching
After photobleaching
After photoactivation
Far-red (654-870 nm)
Red (572-624 nm)
c
Atto 647N
Before photobleaching
After photobleaching
After photoactivation
Far-red (654-870 nm)
Red (572-624 nm)
d
Atto 655
Before photobleaching
After photobleaching
After photoactivation
Far-red (654-870 nm)
Red (572-624 nm)
e
CF633
Before photobleaching
After photobleaching
After photoactivation
Far-red (654-870 nm)
Red (572-624 nm)
f
CF647
Before photobleaching
After photobleaching
After photoactivation
Far-red (654-870 nm)
Red (572-624 nm)
g
CF660C
Before photobleaching
After photobleaching
After photoactivation
Far-red (654-870 nm)
Red (572-624 nm)
h
CF660R
Before photobleaching
After photobleaching
After photoactivation
Far-red (654-870 nm)
Red (572-624 nm)
i
Cy5
Before photobleaching
After photobleaching
After photoactivation
Far-red (654-870 nm)
Red (572-624 nm)
j
Cy5.5
Before photobleaching
After photobleaching
After photoactivation
Far-red (654-870 nm)
Red (572-624 nm)
k
DL650
Before photobleaching
After photobleaching
After photoactivation
Far-red (654-870 nm)
Red (572-624 nm)
l
DY649P1
Before photobleaching
After photobleaching
After photoactivation
Far-red (654-870 nm)
Red (572-624 nm)
m
DY654
Before photobleaching
After photobleaching
After photoactivation
Far-red (654-870 nm)
Red (572-624 nm)
n
SiR
Before photobleaching
After photobleaching
After photoactivation
Far-red (654-870 nm)
Red (572-624 nm)
o
Cy3B
Before photobleaching
After photobleaching
After photoactivation
Red (572-624 nm)
Yellow (500-549 nm)

Supplementary Fig. 4 Blue-conversion of organic dyes *in vivo*

a-n, TIRF images of BG-A647-, BG-A680-, BG-Atto 647N-, BG-Atto 655-, BG-CF633-, BG-CF647-, BG-CF660C-, BG-CF660R-, BG-Cy5-, BG-Cy5.5-, BG-DL650-, BG-DY649P1-, BG-DY654-, and BG-SiR-labeled SNAP-EGFR on COS7 cells in the far-red channel excited at 642 nm at 0.2 W/cm^2 for 500 ms (upper panels) and the red channel excited at 561 nm at 10-20 W/cm^2 for 500 ms (lower panels) before (left panels) and after (middle panels) photobleaching of the dyes (see the details in Methods), followed by photoactivation (right panels) with 405-nm laser illumination at 0.6 W/cm^2 for 20 s. **o,** BG-Cy3B-labeled SNAP-EGFR in the red channel excited at 561 nm (upper panels) and the yellow channel (500-549 nm) excited at 488 nm (lower panels). Scale bars, 5 µm. SNAP-EGFR expressed on COS7 cells was labeled using each organic dye conjugated with the BG moiety. Then, the labeled cells were imaged in both the far-red and the red channels. The labeled dyes were almost completely photobleached by the epi-illumination of the 642-nm laser. All the labeled dyes exhibited a significant level of blue-conversion, except for BG-Atto 655 (only an oxazine dye among the tested dyes), which likely involves the blue-conversion-induced cleavage between Atto 655 and the BG moiety. A significant amount of Atto 647N was remained even after photobleaching by the strong laser illumination due to its unusual quick recovery to a bright state. The fluorescence of CF633, CF660C, and Cy3B exhibited bleed-though to our red (for CF633, CF660C) and yellow (for Cy3B) channel. Although the excitation and emission spectra of Cy3B overlaps in both our red and yellow channels, those of CF633 and CF660C do not overlap with the red channel. It is likely that CF633 and CF660C contained a small but significant amount of fluorescent impurities, which is consistent with a previous report that some far-red commercial dyes contained the impurities12. Nevertheless, this bleed-through did not interfere to detect their blue-conversion as we measured the fluorescence intensity further increased after photobleaching. The blue-conversion of Cy3B indicates that blue-conversion is not restricted to the far-red dyes. Only a low level of blue-converted fluorescence of DY654 was detected in the red channel because its emission wavelength greatly shifted (166 nm) up to the yellow/green channel after the blue-conversion.

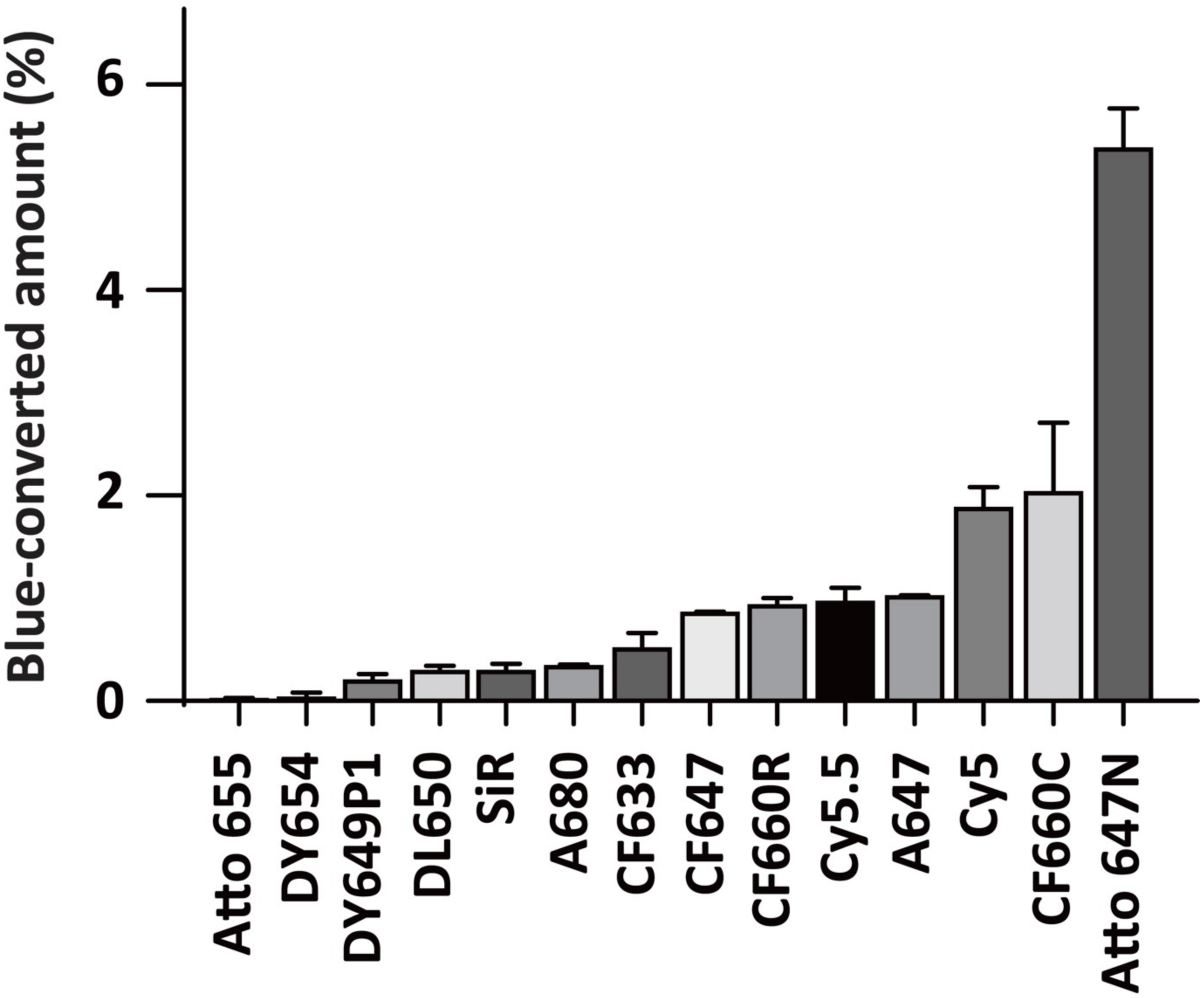

Supplementary Fig. 5 The level of blue-conversion of far-red organic dyes in the red channel

A histogram for the blue-conversion levels of A647, A680, Atto 647N, Atto 655, Cy5, Cy5.5, CF633, CF647, CF660C, CF660R, DL650, DY649p1, DY654, and SiR from the far-red channel to the red channel *in vivo*. The error bars represent the standard error of the mean (SEM) (n > 3).

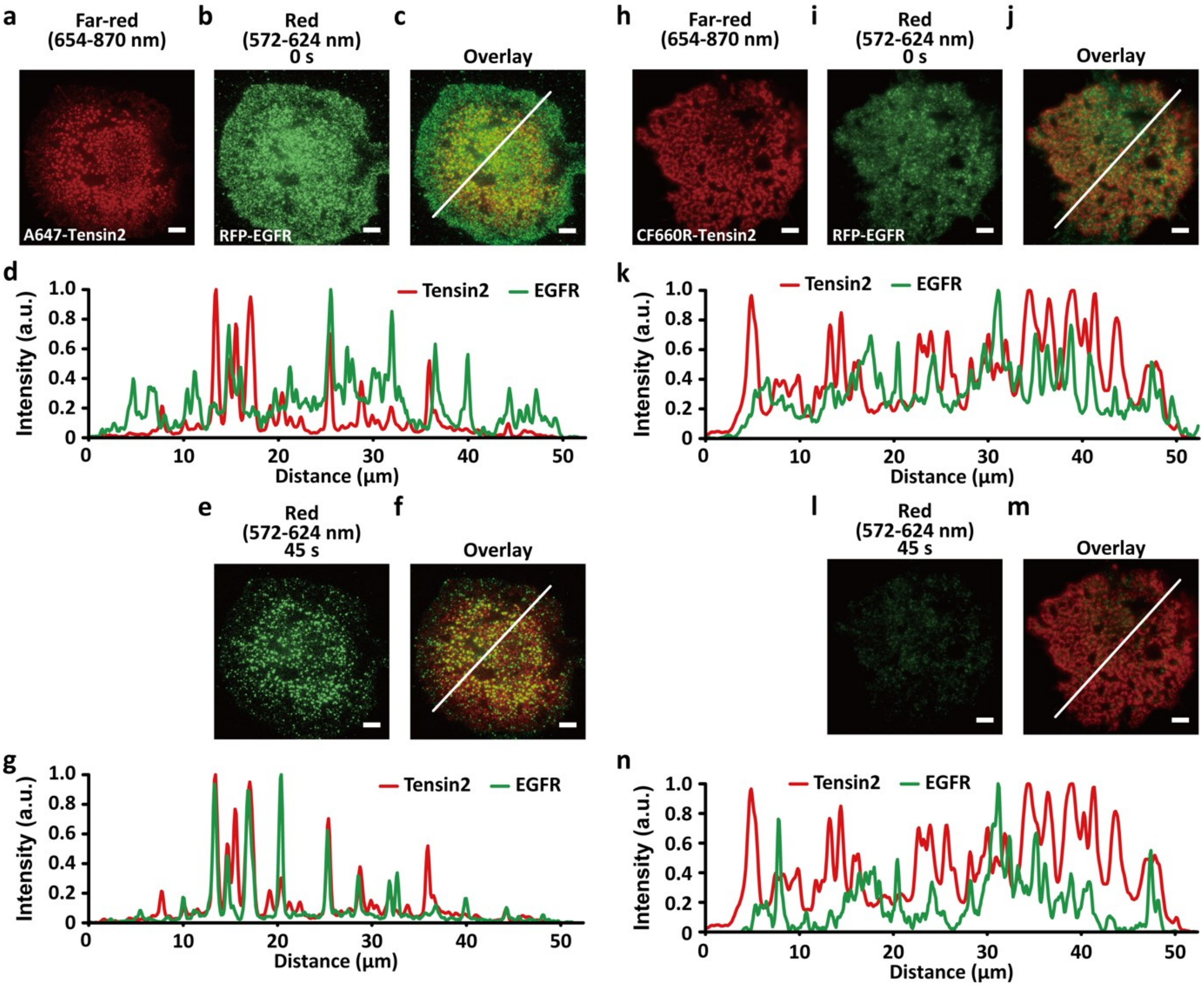

Supplementary Fig. 6 Blue-conversion effect in multicolor colocalization analysis

TIRF images of BG-A647- (**a**) or CF660R- (**h**) labeled SNAP-Tensin2 in the far-red channel. TIRF images of RFP-EGFR in the red channel at 0 s (**b, i**) and at 45 s (**e, l**). Overlays of the images from the two channels (**c, f, j,** and **m**). Intensity profiles of the indicated lines (white) in the overlaid images (**d, g, k,** and **n**). The strong correlation of fluorescence between the two channels was observed along with the indicated line due to the blue-converted fluorescence of A647 after the imaging for 45 s in the red channel, which was not observed at the first image taken at 0 s (**f, g**). No spatial correlation of fluorescence was observed at 0 s and 45 s when CF660R was used to label SNAP-Tensin2 (**m, n**). Scale bars, 5 μm.

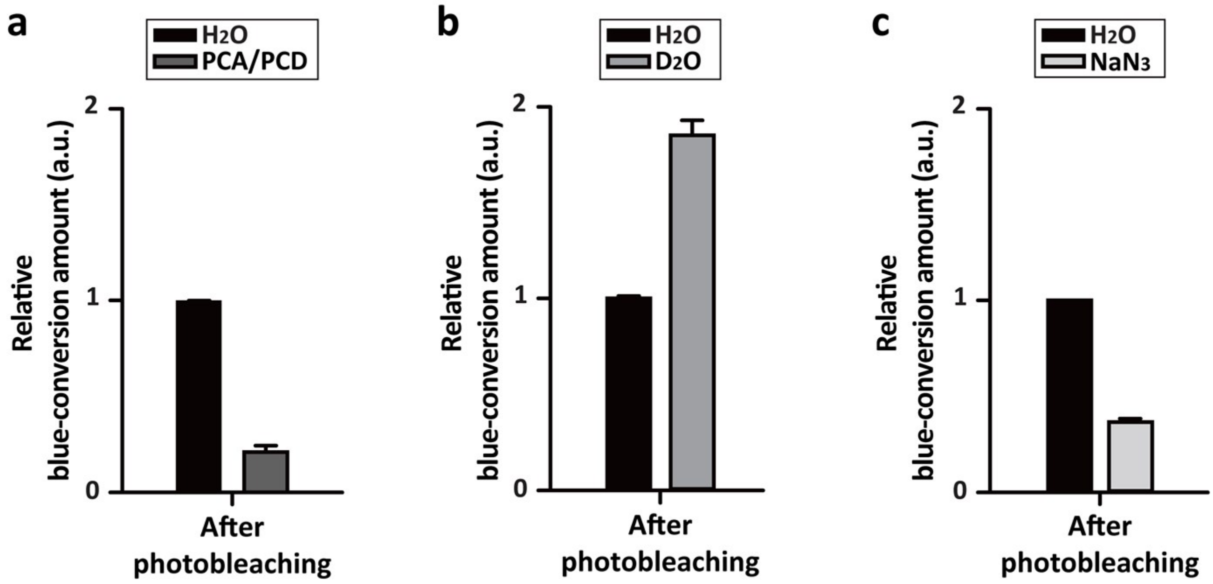

Supplementary Fig. 7 Oxygen, in particular singlet oxygen, is critical to blue-conversion

The involvement of photooxidation in the blue-conversion of A647 was examined. The oxygen was deprived by using the oxygen scavenging system utilizing 5 mM protocatechuic acid (PCA) and 0.5 U/mL protocatechuate-3,4-dioxygenase (PCD). In the presence of PCA/PCD, the blue-conversion level of A647 was significantly reduced (**a**). To examine if a singlet oxygen is involved in the blue-conversion of A647, 45% deuterium oxide (D_2O) that increases a singlet oxygen lifetime or 33 mM sodium azide (NaN_3) that scavenges singlet oxygen. The blue-conversion level of A647 became elevated in the presence of D_2O (**b**) or lowered in the presence of NaN_3 (**c**). The results indicated that the blue-conversion of A647 requires a photooxidative reaction involving singlet oxygen. The error bars represent the standard error of the mean (SEM) (n > 3).

Supplementary Information references

1. E. M. Stennett, M. A. Ciuba and M. Levitus, *Chemical Society Reviews*, 2014, **43**, 1057-1075.

2. T. E. McCann, N. Kosaka, Y. Koide, M. Mitsunaga, P. L. Choyke, T. Nagano, Y. Urano and H. Kobayashi, *Bioconjugate chemistry*, 2011, **22**, 2531-2538.

3. J. C. Vaughan, G. T. Dempsey, E. Sun and X. Zhuang, *Journal of the American Chemical Society*, 2013, **135**, 1197-1200.

4. CF DYES: Next Generation Fluorescent Dyes, https://biotium.com/technology/cf-dyes/, (accessed May 2021)

5. DY-649P1, https://dyomics.com/en/products/red-excitation/dy-649p1, (accessed May 2021).

6. R. Wirth, P. Gao, G. U. Nienhaus, M. Sunbul and A. Jäschke, *Journal of the American Chemical Society*, 2019, **141**, 7562-7571.

7. ATTO 647N, https://www.atto-tec.com/product_info.php?language=en&info=p114_atto-647n.html, (accessed May 2021)
